# Bacterial Microbiome in Wild-Caught *Anopheles* Mosquitoes in Western Thailand

**DOI:** 10.3389/fmicb.2020.00965

**Published:** 2020-05-21

**Authors:** Krajana Tainchum, Chloé Dupont, Theeraphap Chareonviriyaphap, Estelle Jumas-Bilak, Michael J. Bangs, Sylvie Manguin

**Affiliations:** ^1^Agricultural Innovation and Management Division, Faculty of Natural Resources, Prince of Songkla University, Songkhla, Thailand; ^2^Center for Advanced Studies for Agriculture and Food, KU Institute for Advanced Studies, Kasetsart University, Bangkok, Thailand; ^3^HydroSciences Montpellier, Institut de Recherche pour le Développement, CNRS, Université Montpellier, Montpellier, France; ^4^Centre Hospitalier Universitaire, Laboratoire d’Hygiène Hospitalière, Montpellier, France; ^5^Public Health & Malaria Control, PT Freeport Indonesia/International SOS, Kuala Kencana, Indonesia

**Keywords:** *Anopheles* mosquitoes, malaria, bacterial microbiota, biodiversity, Thailand

## Abstract

Among the complex microbial community living in the mosquito midgut, some bacteria (e.g., *Enterobacter* spp.) can deliver effector molecules with anti-*Plasmodium* effects suppressing the development of malaria parasites (*Plasmodium falciparum*) before the öokinete can penetrate the mosquito midgut epithelium. Despite knowledge of this phenomenon, only a few studies have defined the diversity of microbiota in wild-caught adult *Anopheles* species. The objective of this study was to analyze and compare the bacterial microbiota in different *Anopheles* species, including representatives of the primary malaria vectors in western Thailand. Wild female *Anopheles* species were sampled from malaria-endemic areas in Tak and Mae Hong Son provinces near the Thai-Myanmar border. Midgut/abdominal bacterial diversity was assessed by examining the 16S rRNA gene, V3 hypervariable region, using PCR-Temporal Temperature Gel Electrophoresis (PCR-TTGE) profiling and sequence analysis. A total of 24 bacterial genera were identified from eight *Anopheles* species. Five bacterial genera were newly reported in *Anopheles* mosquitoes (*Ferrimonas*, *Megasphaera*, *Pectobacterium*, *Shimwellia*, and *Trabulsiella*). Five genera, including *Megasphaera*, were detected exclusively in a single-malaria (*Plasmodium vivax*) infected *Anopheles minimus* and not observed in other non-infected mosquitoes. The use of PCR-TTGE provides the first characterization of the midgut bacterial microbiome present in wild adult *Anopheles* in Thailand. Evidence that microbiota might impact pathogen development (suppression) in *Anopheles* and thereby reduce the risk of pathogen transmission deserves more studies to describe the presence and better understand the biological role of bacteria in natural mosquito populations.

## Introduction

Despite significant progress in the control of malaria throughout the country, Thailand remains malaria-endemic, particularly along the international borders with Cambodia, Lao PDR, Malaysia, and Myanmar (DDC, 2018). The vast majority of recorded malaria cases, primarily *Plasmodium vivax* (73%) and *Plasmodium falciparum* (18%), occur along the Thai-Myanmar border (70–80%), especially in Tak and Mae Hong Son provinces (DDC, 2018). Of the 79 recognized *Anopheles* species present in Thailand, the most important malaria vectors include two sibling species in the Dirus Complex (*Anopheles baimaii* Sallum & Peyton, and *Anopheles dirus* Peyton & Harrison), *Anopheles minimus* Theobald, *Anopheles aconitus* Dönitz, and three members of the Maculatus Group (*Anopheles maculatus* Theobald, *Anopheles pseudowillmori* [Theobald], and *Anopheles sawadwongporni* Rattanarithikul & Green) (Tananchai et al., 2019).

The malaria sporogonic cycle begins when a female mosquito ingests infective-stage (gametocytes) *Plasmodium* parasites from the blood of an infected human host. The ingested parasites undergo syngamy to create motile stage öokinetes that penetrate the mosquito midgut to become oocysts. The oocyst gradually enlarges as parasites multiply to produce many sporozoites. Once released from the oocyst, sporozoites migrate to the salivary glands to await transmission to another host via a mosquito bite. Although many *Plasmodium* gametocytes can be ingested during a single blood-feeding, only a small fraction typically develop to form oocysts (Leroy et al., 2014). The reason for this sharp decrease in potential infection density is multifactorial (genetic and non-genetic) and in large part influenced by the parasite-vector species relationship and individual mosquito susceptibility (competence) to *Plasmodium* infection allowing successful sporogonic development of the parasite while minimizing significant adverse effects on mosquito fitness (survival, fecundity, etc.) (Black and Severson, 2005; Lefevre et al., 2013). One area of research on this relationship has been the modulating effects linked to certain naturally occurring microbiota in the midgut and abdomen of mosquitoes that can suppress or prevent *Plasmodium* development (Cirimotich et al., 2011b). In particular, the role of enterobacteria that influences parasite development and transmission has been investigated in *Anopheles* mosquitoes. Additional investigations on this phenomenon may help to develop novel methods involving bacterial symbionts to arrest malaria from vector to host.

Symbiotic bacteria, such as *Pantoea agglomerans* and *Asaia* spp., have been successfully transformed to express anti-malaria molecules (anti-plasmodia effector proteins) that render host mosquitoes refractory to malaria infection (Favia et al., 2008; Damiani et al., 2010; Wang et al., 2012); in effect, becoming a paratransgenic means for preventing malaria transmission (i.e., transmission-blocking strategy) (Doumbo et al., 2018). Engineered *P. agglomerans* strains can inhibit up to 98% of *P. falciparum* development in infected mosquitoes (Riehle et al., 2007). *Enterobacter* (Esp_Z) is shown to inhibit öokinete, oocyst, and sporozoite formation of *P. falciparum* in *Anopheles gambiae* by up to 99% (Cirimotich et al., 2011b). Co-infections with *Serratia marcescens* and *P. vivax* in *Anopheles albimanus* have resulted in only 1% of mosquitoes being able to develop oocysts and complete sporogonic development (Minard et al., 2013). In another study, *Serratia marcescens* Y1 strain isolated from field-collected female *Anopheles*, induced anti-plasmodia factors that activated the immune system in *Anopheles stephensi* effectively rendering the mosquito resistant to *Plasmodium berghei* infection (Bai et al., 2019).

The bacterial biodiversity in nine species of field-collected *Anopheles* in Thailand and Vietnam demonstrated complex microbiota in the mosquito midgut and abdomen, primarily Gram-negative bacterial rods, including *Serratia marcescens*, *Klebsiella ozaenae*, *Pseudomonas aeruginosa*, *Escherichia coli*, and *Enterobacter* spp. (Manguin et al., 2013). Other studies have reported the majority of adult mosquito midgut microbiota were Gram-negative species in the phylum *Proteobacteria* (Tandina et al., 2016; Zoure et al., 2020). At least three mosquito-specific bacterial species have been isolated from the midgut of African malaria vectors in the Gambiae Complex, including *Thorsellia anophelis*, *Janibacter anophelis* (Kampfer et al., 2006) and *Elizabethkingia anophelis* (Kampfer et al., 2011). Despite a large amount of work done on malaria vectors over many decades, few studies have examined the natural diversity of microbiota in adult mosquitoes (Manguin et al., 2013; Bassene et al., 2018). Therefore, the objective of this study is to use a sensitive molecular method to evaluate the natural bacterial diversity in wild-caught adult *Anopheles* in a malaria-endemic region of western Thailand.

## Materials and Methods

### Ethics Statement

Human use protocol (human-landing collections) for this study was reviewed and approved by the Ethical Research Committee, Chulalongkorn University, Bangkok, Thailand (No. 0961/56).

### Mosquito Collections and Species Identification

**FIGURE 1:**
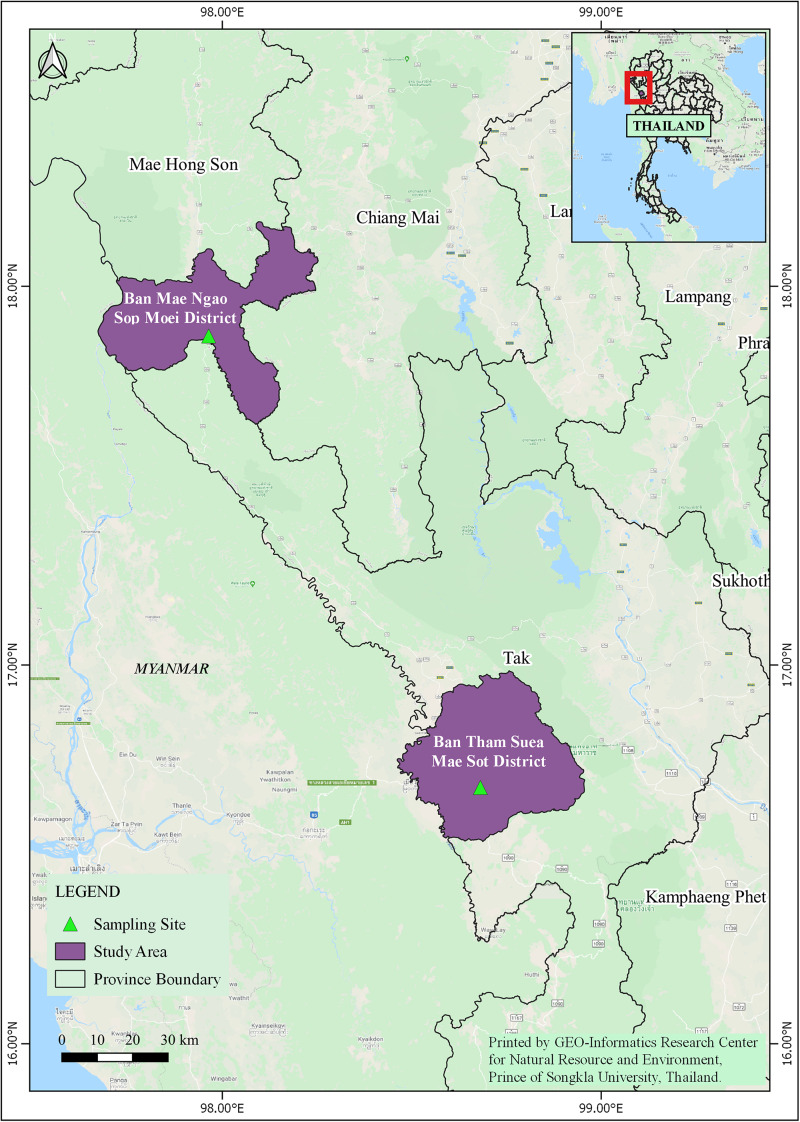
Study site locations in Mae Sot District (Tak Province) and Sop Moei District (Mae Hong Son Province), western Thailand.

**FIGURE 2:**
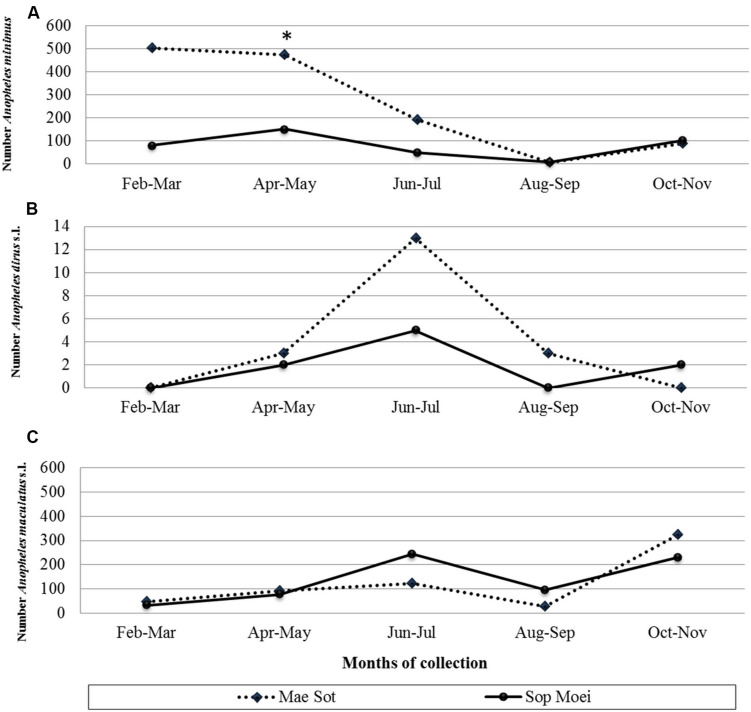
Number of **(A)** *Anopheles minimus* (scale from 0 to 600 specimens), **(B)** *An. dirus* complex (scale from 0 to 14 specimens), and **(C)** *An. maculatus* group (scale from 0 to 600 specimens) mosquitoes collected by location and month (February to November 2011). *, month (April) and location (Mae Sot) where the vivax-infected *An. minimus* was collected.

In 2011, adult *Anopheles* mosquitoes were collected from two hypoendemic malaria locations along the Thai-Myanmar border in Tak and Mae Hong Son provinces, respectively (Figure 1), using a standard human-landing collection technique (Tainchum et al., 2014). Table 1 provides geographic locations, prevailing climatic factors, environment biotype, and crude malaria incidence rate (2010–2012). The abundance of the primary malaria vectors, *An. minimus*, *An. dirus* complex, and *An. maculatus* group, by month of collection (from February to November 2011) and location, is presented in Figure 2. Live mosquitoes were initially sorted using discriminating morphological criteria for species, complex or group level identification (Rattanarithikul et al., 2006). For further mosquito identification, *Anopheles* specimens were assayed using the appropriate allele-specific PCR technique by species complex or group (Tainchum et al., 2014). Each mosquito was divided in two parts, head-thorax for mosquito species identification and detection of malaria (*Plasmodium*) infection, and abdomen retained for bacteriological analysis. Abdomens were stored at −80°C until further processing.

**TABLE 1:** Background information on sampled locations with malaria transmission.

Location	Province	Tak	Mae Hong Son
	District	Mae Sot	Sop Moei
	Subdistrict	Phra That Pha Daeng	Mae Suat
	Village	Ban Tham Suea	Ban Mae Ngao
	GPS coordinates	16°67′ N, 98°68′ E	17°51′ N, 97°57′ E
	Altitude (meter above sea level)	196 m	157 m
**Climatic parameters (diel range)**	Temperature (°C)	15.5 to 36.9	15.7 to 33.5
	Humidity (% RH)	76.5 to 95.0	79.8 to 95.3
	Mean annual rainfall (mm)	2,053	2,060
**Environment**	Main vegetation type	Mixed forest/agriculture	Native forest
**Total population (2010)**		525,684	284,138
Number of reported malaria cases in province per 100,000 population*	2010	1,608.3	569.1
	2011	1,027.2	548.9
	2012	765.3	438.2

*Bureau of Vector-Borne Disease, Ministry of Public Health, Nonthaburi, Thailand. Available source: https://apps.boe.moph.go.th/boeeng/annual.php.

### *Plasmodium* Infection in Mosquitoes

*Plasmodium* detection used an aliquot of the head-thorax DNA extraction used for *Anopheles* species identification. A Roche LightCycler480 (Software Version LCS480 1.5.0.39) was performed for real-time PCR with TaqMan reagents and hydrolysis probes for the detection of *P. falciparum*, *P. vivax*, and *P. knowlesi*, following a slightly modified methodology of Divis et al. (2010).

### DNA Extraction From Abdominal Contents

Each abdomen was rinsed twice in purified water (from sealed injectable solution) before complete disruption using a tissue crusher device in 150 μL of TE buffer. DNA was extracted using the Master Pure Gram Positive DNA purification kit following supplier instructions (Epicentre Biotechnologies, Madison WI, United States).

### Polymerase Chain Reaction

For each sample, the V2–V3 region of the 16S rRNA bacteria gene was amplified using the primers HDA1/HDA2 (Roudière et al., 2009); HDA1: 5′-ACTC CTA CGG GAG GCA GCA GT-3′, HDA2: 5′-GTA TTA CCG CGG CTG CTG GCA-3′. A 40-bp clamp, named GC (5′-CGC CCG GGG CGC GCC CCG GGC GGG GCG GGG GCA CGG GGG G-3′) flanked the 5′ extremity of HDA1 (Ogier et al., 2002) to form HDA1-GC. PCR was performed using an Eppendorf thermal cycler^®^ (Eppendorf, Le Pecq, France) in sterile 0.5 ml tubes. The reaction mixture (50 μL) contained 2.5 units of Taq DNA Polymerase (FastStart High Fidelity PCR System, Roche, Meylan, France), 0.2 mM of each primer and 1 μl of DNA in the appropriate reaction buffer. The amplification cycle was 95°C for 2 min, 35 cycles at 95°C for 1 min, 62°C for 30 s, 72°C for 1 min, and 7 min at 72°C for the final extension step. Solutions were prepared using sterile DNA-free water with the addition of template DNA and gel electrophoresis of PCR products carried out in separate rooms to avoid any possible contamination. PCR amplification of DNA was detected by 2% agarose gel electrophoresis containing ethidium bromide and visualized under ultraviolet light.

### Temporal Temperature Gel Electrophoresis (TTGE) Migration

Temporal temperature gel electrophoresis was performed using the DCode universal mutation detection system (Bio-Rad Laboratories, Marne-la-Coquette, France) with 16 cm × 16 cm × 1 mm gels (Roudière et al., 2009). The 60 ml gels were composed of 8% (w/v) bisacrylamide (37.5:1), 7 M urea, 60 μl of *N,N,N*′,*N*′-tetramethylethylenediamine (TEMED), and 0.1% (w/v) ammonium persulfate. Gels were run with 1X Tris-acetate-EDTA buffer at pH 8.4. A 5 μl PCR product was loaded on the gel with 5 μl in-house dye marker (50% saccharose 50%, 0.1% bromophenol blue) using capillary tips. Denaturing electrophoresis was performed at 46 V with a temperature ramp from 63 to 70°C during 16 h (increment 0.4°C/h) following a pre-migration step of 15 min at 20 V and 63°C. Gels were stained with ethidium bromide solution (5 μg/ml) for 20 min, washed with de-ionized water, and viewed using a UV transillumination system (Vilber Lourmat Sté, France) and photographed.

### TTGE Band Sequencing and OTU Identification

On each TTGE gel, approximately 50% of the bands were sequenced, while other bands were assigned to an affiliated operational taxonomic unit (OTU) by comparing their migration distance with that of sequenced bands. TTGE bands were excised and DNA eluted with 50 μl of elution buffer using Qiaquick PCR purification kit (Qiagen, Courtaboeuf, France) overnight at 37°C before PCR amplification with HDA1/HDA2 without GC clamp. The reaction conditions were identical to those described above. PCR products were sequenced using an ABI 3730xl sequencer (Cogenics, Meylan, France). Each sequencing chromatograph was visually inspected and corrected as appropriate.

The sequences were quality-checked using the SEQMATCH program in the 16S rDNA-specialized database, Ribosomal Database Project (RDP^1^). The 16S rDNA sequences were analyzed using the Basic Local Alignment Search Tool (BLAST) from the GenBank database^2^ and/or the RDP (Cole et al., 2005), for initial sample identifications. The reference sequence with the highest percentage was used for OTU affiliation. Clustered OTUs are based on 97% DNA sequence identity threshold of the 16S gene sequences to distinguish between bacteria at the genus level. A sequence was affiliated to a species-level OTU when the percent of sequence similarity was > 99% (as proposed by Drancourt et al., 2000). This value is above the recognized cut-off value standard for the delineation of species (Stackebrandt and Goebel, 1994), but warrants high stringency for species-level OTU affiliation. Below 99% similarity, the sequence is affiliated to the genus of the reference sequence having the highest percentage. When different species reference sequences match (or near equal), affiliation was done to the genus level. For example, 99.5% sequence similarity between species *Aeromonas caviae* and *Aeromonas hydrophila* was only assigned to the genus *Aeromonas*. Low cut-off is not defined for the genus delineation as affiliation to a higher taxonomic rank, e.g., family or order, was to be done considering the taxonomic frame of the clade using Greengenes database (McDonald et al., 2012).

### Statistical Analysis

The OTU means between the two different regions were compared using a Mann–Whitney U-test for the non-parametric test with the statistical significance level set at *p* < 0.05 using SPSS for Windows version 16 (Chicago, IL, United States).

## Results

### Bacteria in *Anopheles* and Diversity Index

A total of 190 *Anopheles* specimens, representing eight *Anopheles* species, were collected from Mae Sot (Tak Province) and Sop Moei (Mae Hong Son Province) (Table 1 and Figure 1). The peak collection numbers of mosquitoes (*An. minimus*, *An. dirus* complex, and *An. maculatus* group) occurred during the same periods in both locations but differed by species (Figure 2). For instance, in Mae Sot *An. minimus* had higher captures during February and April, while a more modest increase was seen in Sop Moei in May. *Anopheles dirus* s.l. peaked in June and July in Mae Sot and Sop Moei, respectively. For *An. maculatus* s.l., between both locations greater numbers were captured in June-July and October-November timeframes compared to other months (Figure 2). Within the Minimus Complex, only *An. minimus* was identified in association with very few specimens (*n* = 2) of a closely related species, *An. aconitus* (Funestus Group) (Table 2). Two species in the Dirus Complex, *An. dirus* and *An. baimaii*, and four Maculatus Group species, *An. maculatus*, *An. sawadwongporni*, *An. pseudowillmori* and *An. dravidicus* were molecularly identified (Table 2).

**TABLE 2:** Sample number and species of *Anopheles* assayed for abdominal bacteria, those found PCR-TGGE bacteria positive, and bacteria designated either Gram-negative or Gram-positive.

Collection sites	Mae Sot					Sop Moei				
		
*Anopheles* species	*N*	No. positive by PCR-TTGE	No. sequences	Gram-negative	Gram-positive	*N*	No. positive by PCR-TTGE	No. sequences	Gram-negative	Gram-positive
*An. minimus*	23	13	30	20	3	12	8	12	9	−
*An. aconitus*	1	−	−	−	−	1	−	−	−	−
*An. maculatus*	23	4	10	7	−	5	2	2	2	−
*An. sawadwongporni*	7	2	4	3	−	12	8	10	9	−
*An. pseudowillmori*	1	−	−	−	−	34	4	6	4	1
*An. dravidicus*	5	1	1	1	−	1	−	−	−	−
*An. dirus*	19	5	15	7	−	8	3	9	4	−
*An. baimaii*	25	4	6	4	0	13	2	2	1	−
	104	29	66	42	3	86	27	41	29	1
Total*	190	56	107	71	4					

*Total for both study sites, Mae Sot and Sop Moei.

**FIGURE 3:**
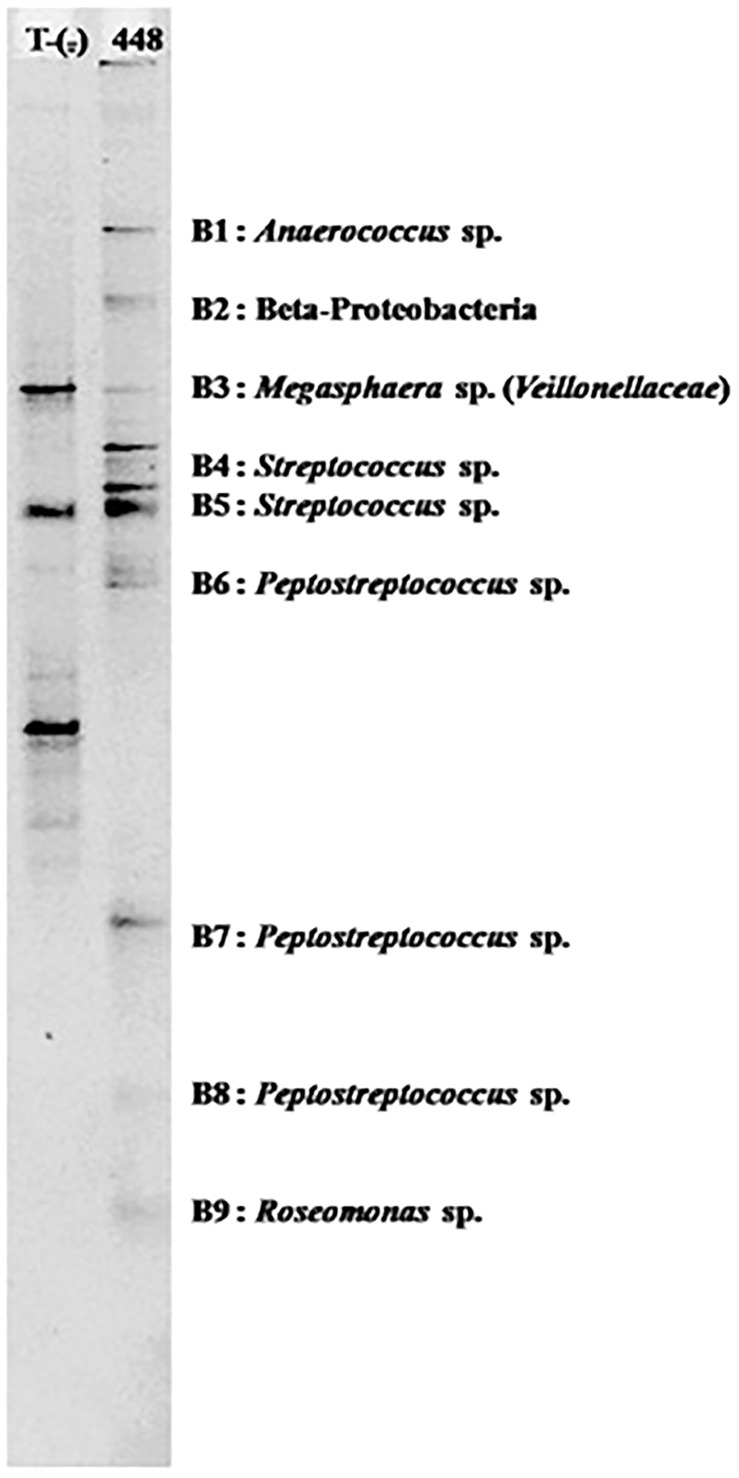
PCR-TTGE banding patterns for bacterial genera detected in the abdomen of one naturally *Plasmodium vivax* infected *An. minimus*.

From 104 *Anopheles* assayed from Mae Sot and 86 from Sop Moei, only one (0.53%) mosquito, *An. minimus*, was found with malaria (*P. vivax*) sporozoites. This specimen was collected in Mae Sot (Tak Province) in April 2011 during the typical warm-dry season (Figure 2). The abdominal microbiota findings of all 190 assayed specimens, including the malaria-infected sample (Figure 3), was based on isolating the 16S rRNA gene using PCR-TTGE. In total, 107 sequences were obtained from 56 mosquitoes (30% of total sample) (Table 2).

**FIGURE 4:**
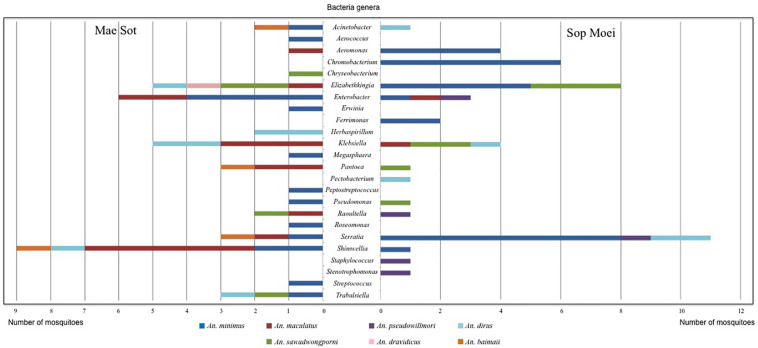
Bacterial genera identified in *Anopheles* species by PCR-TTGE.

A raw diversity index that globally reflects the bacterial diversity in a sample is typically reflected by counting the resulting bands in TTGE profiles. The number of bands ranged from one to 10, suggesting that the bacterial diversity per mosquito also ranged from one to 10 OTUs. However, subsequent sequencing showed that bands with different migration distances could belong to the same OTU. This atypical phenomenon was observed for bacteria displaying sequence heterogeneity among the 16S rRNA gene copies. For example, most members of genera in the large family *Enterobacteriaceae* displayed a high level of 16S rRNA gene heterogeneity, thus producing complex banding patterns. Considering that *Enterobacteriaceae* were relatively common in our samples, the raw diversity index overestimated the actual bacterial diversity. Therefore, a refined diversity index was calculated after affiliation of each band to an OTU by sequencing or by a comparative analysis approach. The resulting index showed a bacterial diversity with an average of 1.7 OTU per specimen. The number of OTUs per specimen did not differ between mosquitoes in the two locations, with an average OTU of 1.69 and 1.7 per specimen in Mae Sot and Sop Moei, respectively (*p* = 0.345). *Anopheles minimus* hosted the majority of OTU identified in this study, 50% and 29% of OTU identified in Mae Sot and Sop Moei, respectively; however, it was also the most abundant *Anopheles* species captured (Figure 4).

### Bacterial Diversity in *Anopheles* Mosquitoes in Western Thailand

The 16S rRNA gene PCR-TTGE that focused on the hypervariable V3 region produced sequences of approximately 200 bp, which are generally of size not informative enough for species-specific affiliation. Consequently, this study presents the bacterial diversity at the genus level. However, probable species affiliation was proposed for several genera when the phylogenetic signal of the V3 region was regarded significant. Contrasting with the low diversity per individual (average 1.7), collectively the OTU diversity in the entire sampling was high with 24 different bacterial genera distributed among three phyla; *Proteobacteria* (71%), *Firmicutes* (21%), and *Bacteroidetes* (8%) (Table 3). *Proteobacteria*, the predominant microbiota, encompassed the Alpha, Beta, and Gamma superclasses.

**TABLE 3:** Bacterial genera detected in adult *Anopheles* abdomens collected in Mae Sot and Sop Moei districts, western Thailand.

Bacterial genera (number of *Anopheles* species)	Bacterial phyla	*An. minimus*		*An. dirus*		*An. baimaii*		*An. maculatus*		*An. sawadwongporni*		*An. dravidicus*		*An. pseudowillmori*	
								
		M^a^	S^b^	M	S	M	S	M	S	M	S	M	S	M	S
		(*n* = 13)	(*n* = 8)	(*n* = 5)	(*n* = 3)	(*n* = 4)	(*n* = 2)	(*n* = 4)	(*n* = 2)	(*n* = 2)	(*n* = 8)	(*n* = 1)	(*n* = 0)	(*n* = 0)	(*n* = 4)
*Acinetobacter* (3)	*Proteobacteria*	1	−	−	1	1	−	−	−	−	−	−	−	−	−
*Aerococcus*# (1)	*Firmicutes*	1	−	−	−	−	−	−	−	−	−	−	−	−	−
*Aeromonas* (2)	*Proteobacteria*	−	4	−	−	−	−	1	−	−	−	−	−	−	−
*Chromobacterium* (1)	*Proteobacteria*	−	6	−	−	−	−	−	−	−	−	−	−	−	−
*Chryseobacterium* (1)	*Bacteroidetes*	−	−	−	−	−	−	−	−	1	−	−	−	−	−
*Elizabethkingia* (5)	*Bacteroidetes*	−	5	1	−	−	−	1	−	2	3	1	−	−	−
*Enterobacter* (3)	*Proteobacteria*	4	1	−	−	−	−	2	1	−	−	−	−	−	1
*Erwinia* (1)	*Proteobacteria*	1	−	−	−	−	−	−	−	−	−	−	−	−	−
Ferrimonas* (1)	*Proteobacteria*	−	2	−	−	−	−	−	−	−	−	−	−	−	−
*Herbaspirillum* (1)	*Proteobacteria*	−	−	2	−	−	−	−	−	−	−	−	−	−	−
*Klebsiella* (3)	*Proteobacteria*	−	−	2	1	−	−	3	1	−	2	−	−	−	−
Megasphaera*# (1)	*Firmicutes*	1	−	−	−	−	−	−	−	−	−	−	−	−	−
*Pantoea* (3)	*Proteobacteria*	−	−	−	−	1	−	2	−	−	1	−	−	−	−
Pectobacterium* (1)	*Proteobacteria*	−	−	−	1	−	−	−	−	−	−	−	−	−	−
*Peptostreptococcus*# (1)	*Firmicutes*	1	−	−	−	−	−	−	−	−	−	−	−	−	−
*Pseudomonas* (2)	*Proteobacteria*	1	−	−	−	−	−	−	−	−	1	−	−	−	−
*Raoultella* (3)	*Proteobacteria*	−	−	−	−	−	−	1	−	1	−	−	−	−	1
*Roseomonas*# (1)	*Proteobacteria*	1	−	−	−	−	−	−	−	−	−	−	−	−	−
*Serratia* (5)	*Proteobacteria*	1	8	−	2	1	−	1	−	−	−	−	−	−	1
Shimwellia* (4)	*Proteobacteria*	2	1	1	−	1	−	5	−	−	−	−	−	−	−
*Staphylococcus* (1)	*Firmicutes*	−	−	−	−	−	−	−	−	−	−	−	−	−	1
*Stenotrophomonas* (1)	*Proteobacteria*	−	−	−	−	−	−	−	−	−	−	−	−	−	1
*Streptococcus*# (1)	*Firmicutes*	1	−	−	−	−	−	−	−	−	−	−	−	−	−
Trabulsiella* (3)	*Proteobacteria*	1	−	1	−	−	−	−	−	1	−	−	−	−	−

*Five genera or OTU newly identified in *Anopheles* mosquitoes. ^#^Five bacterial genera only detected from one *Anopheles minimus* infected with *Plasmodium vivax*. ^*a*^M = Mae Sot (Tak Province); ^*b*^S = Sop Moei (Mae Hong Son Province).

Within the seven *Anopheles* species analyzed (*An. aconitus* excluded), *An. minimus* was more microbiota diverse with 16 bacterial genera identified, followed by *An. dirus* and *An. maculatus*, with half the number of genera (8) detected per species. However, *An. minimus* was a dominant species captured (*n* = 35) and analyzed based on PCR-TTGE (21 specimens out of 56) (Figure 4 and Table 3). Nineteen and 15 genera were identified in Mae Sot and Sop Moei respectively, with 10 shared genera (Figure 4). The comparison of bacterial diversity between the four *Anopheles* species collected at both locations (*An. minimus*, *An. dirus*, *An. maculatus*, and *An. sawadwongporni*) revealed in three instances the number of genera was higher in Mae Sot than Sop Moei.

Among the 24 bacterial genera identified, 14 (58%) were shared by at least two specimens and 10 were identified in only one mosquito (Table 3). Among these 14 genera, two (*Elizabethkingia* and *Serratia*) were shared by five *Anopheles* species out of seven, one (*Shimwellia*) by four species, six (*Acinetobacter*, *Enterobacter*, *Klebsiella*, *Pantoea*, *Raoultella*, *Trabulsiella*) in three species, two (*Aeromonas*, *Pseudomonas*) in two species, and three (*Chromobacterium*, *Ferrimonas*, *Herbaspirillum*) identified in at least two specimens of the same species (Table 3 and Figure 4).

Nine of the 24 genera (37.5%) belonged to *Enterobacteriaceae*, including species within the genus *Enterobacter*, *Erwinia*, *Klebsiella*, *Pantoea*, *Pectobacterium*, *Raoultella*, *Serratia*, *Shimwellia*, and *Trabulsiella* (Table 3). The *Anopheles* microbiota included four primary species, *Serratia marcescens* (*n* = 14), *Shimwellia blattae* (*n* = 10), *Enterobacter cloacae* (*n* = 9), and *Klebsiella pneumoniae* (*n* = 9). *Shimwellia* was the dominant genus identified in Mae Sot (*n* = 9 specimens), while *Serratia* was dominant in Sop Moei (*n* = 11 specimens) (Figure 4). *Elizabethkingia*, which belongs to the *Bacteroidetes* phylum, was the second most common genus identified, present in the abdomen of 13 *Anopheles* specimens among five species (71.5%) – *An. minimus*, *An. dirus*, *An. maculatus*, *An. sawadwongporni*, and *An. dravidicus.* Sequences affiliated with the genus *Elizabethkingia* could not be definitively assigned to either *E. anophelis* or *Elizabethkingia meningoseptica* as the V3 region cannot discriminate between the *Anopheles*-specific bacterium and the bacterium as human pathogen. Among the 10 genera identified in only one *Anopheles* adult, six were found in *An. minimus* (*Aerococcus*, *Erwinia*, *Megasphaera*, *Peptostreptococcus*, *Roseomonas*, and *Streptococcus*). The other four genera were *Staphylococcus* and *Stenotrophomonas* in *An. pseudowillmori, Pectobacterium* in *An. dirus*, and *Chryseobacterium* in *An. sawadwongporni.*

In all, 24 bacterial genera were identified in this study, of which 14 have been reported previously in another study (Manguin et al., 2013). Five genera are newly identified in wild-caught adult *Anopheles*, including *Megasphaera* found only in a malaria-infected *An. minimus* in Mae Sot (Figures 3, 4 and Table 3). In addition, this single malaria-infected mosquito hosted five genera not identified in the remaining non-infected *Anopheles* assayed, including four genera within the phylum *Firmicutes* (*Aerococcus*, *Megasphaera*, *Peptostreptococcus, Streptococcus*) and one genus, *Roseomonas*, in the phylum *Proteobacteria* (Figure 3 and Table 3). Interestingly, three of these five genera are Gram-positive cocci, out of the total four cocci genera identified in this study. Of the 10 genera that were observed only once, half were identified in the only *Plasmodium-*infected mosquito from the 190 sampled (Table 3).

## Discussion

Native adult *Anopheles* species were sampled from malaria-endemic areas in Tak and Mae Hong Son provinces near the Thai-Myanmar border. Bacterial diversity was assessed by the 16S rRNA gene, V3 hypervariable region, PCR-Temporal Temperature Gel Electrophoresis (PCR-TTGE) profiling and sequence analysis. The method provides the first estimation of the abdominal bacterial biodiversity present in field-collected *Anopheles* mosquitoes in Thailand. A total of 24 bacterial genera were identified from eight *Anopheles* species. *Anopheles minimus* presented a higher bacterial diversity than the other sampled *Anopheles* species identified in this study. Five bacterial genera were detected from a single malaria (*P. vivax*) infected *An. minimus*. Five genera are newly reported from field-collected *Anopheles* mosquitoes (*Ferrimonas*, *Megasphaera*, *Pectobacterium*, *Shimwellia*, and *Trabulsiella*), suggesting that the diversity of bacteria in *Anopheles* remains largely underestimated. *Elizabethkingia* spp., a relatively common midgut bacterium in *Anopheles* was also detected. This genus appears to have a role in increasing iron metabolism necessary for bacterial growth and *Plasmodium* development (Clark et al., 2014; Sharma et al., 2019). Bacterial microbiota in *Anopheles* and their effects on the development of *Plasmodium* parasites as a potential means of controlling malaria transmission have been reported in various *Anopheles* species, but the precise mechanisms of activity remain poorly understood (Dong et al., 2009; Meister et al., 2009; Abdul-Ghani et al., 2012; Gendrin and Christophides, 2013; Minard et al., 2013; Hughes et al., 2014; Sharma et al., 2014; Villegas and Pimenta, 2014; Romoli and Gendrin, 2018). The PCR-TTGE described bacteria for 30% of *Anopheles* (56/190) analyzed and appears an efficient tool to investigate bacterial diversity in large samplings of mosquitoes. This method is appropriate to detect bacterial communities with low to moderate diversities as seen in wild-caught *Anopheles* samples in this study and other investigations (Manguin et al., 2013; Ngo et al., 2015, 2016). However, the method presents some limitations due to the restricted number of bands that can be separated within the length of the gel. Optimization of TTGE conditions allows the separation of bands with a minimum distance of 0.1 mm. Therefore, the TTGE procedure would present difficulties to interpret bacterial diversity that exceeds 25 to 30 OTUs per mosquito sample (Roudière et al., 2009).

In this study, the anopheline microbiota displayed TTGE profiles that did not exceed 10 bands; however, the profiles have been interpreted with some difficulty due to inherent heterogeneities in rRNA genes for many bacterial species present in the mosquito ecosystem. At the genomic level, rRNA genes are generally organized in multigene families (Acinas et al., 2004) and sequences show low variability within species, subspecies or genome level (Liao, 2000). That aside, TTGE remains useful by providing an accurate ‘snapshot’ of microbiota in different populations of hosts. Results obtained with TTGE fingerprinting compared to pyrosequencing or Next-Generation Sequencing have demonstrated good correlation for the detection of the majority of OTUs in complex microbiotic communities (Manguin et al., 2013; Li et al., 2014).

Nineteen of 24 bacterial genera detected have been reported from field-collected *Anopheles* (Abdul-Ghani et al., 2012; Gendrin and Christophides, 2013; Minard et al., 2013; Hughes et al., 2014; Sharma et al., 2014; Villegas and Pimenta, 2014). From western Thailand, the abdominal microbiota presented large inter-specimen variability but was dominated by *Serratia*, *Elizabethkingia, Shimwellia*, *Enterobacter* and *Klebsiella*, particularly members in the family *Enterobacteriaceae*. These results demonstrate that PCR-TTGE has fairly low detectability of the minority and/or low-density bacteria populations. This low resolution is a limitation but alternatively can be beneficial for this type of work as the majority of taxa detected by TTGE probably corresponds to true symbiotic endo-colonizers in *Anopheles* and are not likely to be transient or incidental contaminant bacteria.

The presence of enterobacteria is of particular interest because mosquitoes harboring abundant commensal *Enterobacteriaceae* appear more likely to be infected by *P. falciparum* suggesting a possible protective role of these bacteria for natural *Plasmodium* infection (Boissière et al., 2012). On the contrary, some species of *Enterobacteriaceae*, particularly *S. marcescens* and *Enterobacter* (*Esp_Z*), are able to inhibit *Plasmodium* development in *Anopheles* midguts (Gonzalez-Ceron et al., 2003; Cirimotich et al., 2011a; Bai et al., 2019). For instance, *S. marcescens* in *An. albimanus* is associated with inhibition of *P. vivax* oocyst development (Gonzalez-Ceron et al., 2003). In the same phylum (*Proteobacteria*, family *Neisseriaceae*), the presence of *Chromobacterium Csp_P* is associated with a significant reduction in susceptibility of *An. gambiae* to *P. falciparum* (Ramirez et al., 2014).

Members of the genus *Elizabethkingia* detected in assayed mosquitoes from Thailand could not be definitively identified as *E. anophelis* given its close relatedness in the 16S rRNA gene sequence with *E. meningoseptica*. This latter bacteria species is generally widely dispersed in the environment and recognized as an occasional serious bacterial pathogen in humans giving rise to meningitis and pneumonia (Xie et al., 2009). Previous studies reported that *E. anophelis* is a dominant midgut bacterium of laboratory-reared *An. stephensi* and *An. gambiae* (Boissière et al., 2012; Ngwa et al., 2013). Sharma et al. (2019) reported an interesting microbial interaction and immune modulation, whereby the presence of *P. vivax* in *An. stephensi* provoked a metabolic alteration of the availability of iron and nutritional physiology required for ideal bacterial growth resulting in a reduction of the microbiota present in the midgut, particularly the predominant bacteria *Elizabethkingia* and *Pseudomonas*. In addition to the parasite suppressing gut immunity in the mosquito host, by retarding bacterial growth, a greater amount of time is then allowed for the parasite to successfully cross the midgut wall and develop into the oocyst stage.

Within the genus *Staphylococcus*, *S. sciuri* was detected in *An. pseudowillmori*, a bacterium also isolated in the abdomen of other *Anopheles* species in Vietnam, such as *Anopheles crawfordi* and *Anopheles barbumbrosus* (Ngo et al., 2015). *Enterobacter* and *Staphylococcus* are among the genera showing trans-stadial maintenance in *Anopheles* and have been found in *An. albimanus* (*Enterobacter*) and mainly in male *An. stephensi* (*Staphylococcus*) (Rani et al., 2009; Minard et al., 2013; Galeano-Castaneda et al., 2020). Two other genera, *Aerococcus* and *Peptostreptococcus*, were found in *An. minimus* from Thailand, similar to findings from Vietnam (Ngo et al., 2016).

In this study, one *An. minimus* from Mae Sot was found naturally infected with *P. vivax* (Tainchum et al., 2014). This mosquito had at least five genera (*Aerococcus*, *Megasphaera, Peptostreptococcus, Roseomonas, Streptococcus*), bacteria not present in the malaria-free *Anopheles*. Moreover, the malaria-infected mosquito was devoid of *Enterobacteriaceae* and had three of the Gram-positive cocci out of the four total cocci genera detected in the study. Cocci bacteria are commonly present in larval habitats, which might explain their presence in *Anopheles* mosquitoes (Rejmankova et al., 2000; Piyaratne et al., 2005). Although no reasonable extrapolation or conclusion can be drawn from the findings in a single infected mosquito, the larger microbiota diversity found in this specimen is concordant with observations on microbiota detected in African *An. gambiae* s.l. and *Anopheles funestus* in which microbial diversity was greater in *P. falciparum*-infected samples than in non-infected ones (Bassene et al., 2018). Some bacterial symbionts were absent in the infected *An. minimus* specimen, notably *Elizabethkingia* and *Serratia*, which were commonly present in non-infected mosquitoes. Again, the single malaria-infected *Anopheles* was insufficient to allow a comparative analysis of bacterial species biodiversity between malaria-infected and non-infected mosquitoes. One possible limitation of the study design was only focusing on detection of infectious stage sporozoites (post-oocyst) present in the head-thorax portion of the mosquito – the final stage of the *Plasmodium* sporogony. We acknowledge a potential methodology flaw in which additional mosquito infections present as developing oocysts, would have escaped detection. However, having detected *Plasmodium* in the head-thorax indicates that we found sporozoites and the infected mosquito was a malaria vector, while detecting parasite stages in the abdomen is not a guaranty that the mosquito is a vector.

Although, the infected *An. minimus* was collected during a peak abundance period characteristic for this species, malaria-infected mosquitoes are becoming relatively rare findings due to a substantial decrease of malaria transmission in Thailand. A recent study in western Thailand showed *P. vivax*-infected *An. minimus* during an April period with an overall 0.76% sporozoite rate for the Minimus Complex (Tainchum et al., 2014; Sriwichai et al., 2016), comparable to this study (0.53%) and higher than the 0.092% reported by Tainchum et al. (2014). Therefore, in order to investigate and compare the natural microbiota between *Plasmodium*-infected and non-infected *Anopheles*, further collections and analyses are required. The mosquitoes found without *Plasmodium* displayed relatively high enterobacteria diversity, especially the genus *Serratia*. Identification of enterobacteria species in more samples will be the next step in the search for *Enterobacter* (*Esp_Z*) and *Chromobacterium* (*Csp_P*), both known to inhibit *P. falciparum* development (Cirimotich et al., 2011a).

## Conclusion

The analysis of the microbiota detected in the abdomens of eight field-caught *Anopheles* species from Thailand resulted in 24 bacterial genera, among which five were only detected from one *P. vivax*-infected *An. minimus* specimen. A total of five genera were newly reported in *Anopheles* mosquitoes of which *Megasphaera* was only found in the malaria-infected mosquito. *Serratia* and *Elizabethkingia* were the most frequent bacteria found in the anopheline abdomens. Findings of low bacterial diversity, ranging from one to five genera per *Anopheles*, contrasted with a high overall OTU diversity in the entire sampling of species from both localities, which included three major bacterial phyla, *Proteobacteria*, *Firmicutes*, and *Bacteroidetes*. An analysis of the bacterial biodiversity in mosquitoes infected by malaria parasites compared with non-infected specimens was not possible due to the insufficient number of *Plasmodium* infections detected. Five genera identified in the infected specimen were not detected in other specimens, including three Gram-positive cocci. The PCR-TTGE method with bacterial 16S rRNA provided the first estimation of bacterial biodiversity present in *Anopheles* in Thailand. These findings suggest that bacterial diversity in *Anopheles* remains underestimated and requires further investigation. As some microbiota can suppress or block human pathogen development in *Anopheles* vectors, thus reducing the risk of transmission, more studies are needed to better understand the role of naturally occurring bacteria in wild mosquito populations as a potential method of disease control.

## Data Availability Statement

The raw data supporting the conclusions of this article will be made available by the authors, without undue reservation, to any qualified researcher.

## Author Contributions

KT collected the specimens, made the mosquito identification, participated in the bacterial analyses and result’s interpretation, and wrote the draft. CD made the bacterial analyses, their interpretation and improved substantially the manuscript. EJ-B supervised the study design and was involved in the bacterial sequence analyses. TC supervised the mosquito collections and analyses done in Thailand. MB improved the manuscript and participated in the data analyses. SM was at the origin of the study design, was highly involved in data analyses and writing the manuscript. All the authors contributed to the manuscript redaction.

## Conflict of Interest

MB was employed by PT Freeport Indonesia/International SOS. The remaining authors declare that the research was conducted in the absence of any commercial or financial relationships that could be construed as a potential conflict of interest.

## Footnotes

1 http://rdp.cme.msu.edu

2 http://www.ncbi.nlm.nih.gov/blast/
